# Effects of theobromine addition on chemical and mechanical properties of a conventional glass ionomer cement

**DOI:** 10.1007/s40204-019-0107-8

**Published:** 2019-02-06

**Authors:** Fabricio Marcelo Cevallos González, Erika Michele dos Santos Araújo, Maria Regina Lorenzetti Simionato, Luciana Kfouri Siriani, Ana Del Carmen Armas Vega, Igor Studart Medeiros, Adriana Bona Matos

**Affiliations:** 1 Inter Institutional PhD Program at School of Dentistry of University of São Paulo (USP), São Paulo, SP Brazil; 2 School of Dentistry, Central University of Ecuador, Quito, Ecuador; 3 Department of Operative Dentistry, School of Dentistry, University of São Paulo (USP), São Paulo, SP Brazil; 4 Department of Microbiology, Institute of Biomedical Sciences, University of São Paulo (USP), São Paulo, SP Brazil; 5 Department of Operative Dentistry, School of Dentistry, University of São Paulo (USP), São Paulo, Brazil; 6 Department of Biomaterials and Oral Biology, School of Dentistry, University of São Paulo, São Paulo, SP Brazil; 7 Department of Operative Dentistry, School of Dentistry, University of São Paulo (USP), Av. Prof. Lineu Prestes 2227, Cidade Universitária, São Paulo, SP CEP 05508-000 Brazil

**Keywords:** Theobromine, Glass ionomer, Chemical properties, Mechanical properties, Biological properties

## Abstract

In vitro effect of 1% theobromine addition on the physical and chemical properties of conventional glass ionomer (GIC) cement was investigated. Conventional GIC (GIC-C) and 1% theobromine added to GIC (GIC-THEO) specimens were compared regarding the microhardness (*n* = 10), sorption (*n* = 5), solubility (*n* = 5), color change (*n* = 10), fluoride release in saliva (*n* = 10) and the amount of biofilm deposition (*n* = 20). Compared against conventional GIC, adding 1% theobromine increased microhardness (*p* < 0.05), while its sorption, solubility, color and fluoride release to saliva (*p* > 0.05) remained unchanged. On the other hand, *Streptococcus mutans* biofilm amount deposited on its surface decreased statistically when theobromine was added to GIC (*p* < 0.05). Based on the results, it could be concluded that 1% theobromine addition to GIC can be a good strategy as it keeps some of its properties and improves microhardness and biofilm deposits strengthening its role in the preventive approach of dentistry.

## Introduction

Contemporary minimal intervention strategy targets professional care based on early detection of carious lesions and a noninvasive treatment approach. In this context, the use of glass ionomer cement (GIC) as a restorative material, as well as ITR technique (interim therapeutic restorations), plays a fundamental role due to some of its properties, such as ionic bonds between the carboxylic groups of the polyacid molecules and calcium ions of enamel and dentine; anti-cariogenic properties; and fluoride release to the oral environment (Sidhu and Nicholson 2016). The latter not only favors remineralization but also increases enamel and dentine resistance to demineralization, preventing secondary lesions (Hafshejani et al. 2017). On the other hand, other properties, such as microhardness, tensile strength, abrasion, poor polishing and esthetics limit its use (Sidhu and Nicholson 2016; Hafshejani et al. 2017).

Studies have focused on the inclusion of plant- and/or fruit-based agents into restorative materials (Zhao et al. 2014; Amaechi et al. 2013; Osawa et al. 2001) especially in the prevention of caries. In particular, studies reported that *Theobroma cacao,* with its high amounts of flavonoids and polyphenols obtained from both bark and seeds, provides bacteriostatic and remineralizing effects that inhibit biofilm progression by reducing the activity of *Streptococcus mutans (S. mutans)* and consequently decreasing caries (Percival et al. 2006; Amaechi et al. 2013; Zhao et al. 2014).

Studies demonstrate the efficacy of cocoa bean extract in preventing growth and development of cariogenic bacteria in the oral environment, a process that involves the reduction of acid production and glucan synthesis by *S. mutans*, with inhibitory activity in glucosyltransferase (Matsumoto et al. 2004; Zhao et al. 2014). There is also scientific evidence concerning the antioxidant capacity of certain polyphenolic compounds, flavonoids and cocoa trace elements in reducing the inflammatory activity of cytokines (Percival et al. 2006). It is also worth mentioning that other cocoa components, such as oleic and linoleic acids, have antibacterial inhibitory action against *S. mutans* (Ooshima et al. 2000; Osawa et al. 2001). Other in vitro studies reported that alkaloids obtained from cocoa seeds or bark have the ability to remineralize enamel through the formation of apatite and increase in crystal size, creating a harder and acid dissolution-resistant enamel (Kargul et al. 2012; Amaechi et al. 2015; Lippert 2017; Simmons et al. 2016).

In this perspective, theobromine could be considered an additional choice to fluoride compounds, providing greater antibacterial, remineralization or desensitization efficacy (Kargul et al. 2012). When added to dentifrices, theobromine produces apparent dentinal tubules obliteration, which might reduce sensitivity (Amaechi et al. 2015; Lippert 2017).

Thus, due to promising results when added to other dental materials (Osawa et al. 2001; Matsumoto et al. 2004; Percival et al. 2006; Ferrazano et al. 2009; Kargul et al. 2012; Amaechi et al. 2015; Lippert 2017), this in vitro study evaluated the addition of theobromine to GIC in an attempt to increase its anti-cariogenic effects without altering its physical and chemical properties. This innovative material could be established as an additional strategy for caries treatment.

The aim of this study was to evaluate the in vitro effects of the addition of 1% theobromine to conventional glass ionomer cement on microhardness, sorption, solubility, color, and fluoride release in artificial saliva and on biofilm formation by *S. mutans*. The null hypothesis tested was that the addition of theobromine does not interfere in GIC properties, such as microhardness, color, sorption, solubility, antimicrobial ability and fluoride release.

## Materials and methods

In this study, a conventional glass ionomer cement, GIC (Gold Fuji 9, GC Corp, Japan, Lot Number 1701241), was used as control (GIC-C). The experimental compound (GIC-THEO) was obtained by incorporating 1% per weight of theobromine (Sigma-Aldrich, Darmstadt, Germany), weighed on a 0.01 mg precision scale of (ATX 224, Shimadzu, Japan), into the GIC powder. This concentration was used in a pilot study, considering the higher concentration of theobromine incorporated that maintained the shiny appearance of the surface mixture.

### Specimen preparation

Round matrices of three different dimensions (diameter × thickness, in millimeters) were used: Teflon split (8.6 × 1.65) for the fluoride release; aluminum (12 × 1) for biofilm formation; and aluminum (15 × 1) for microhardness, color, sorption and solubility tests.

The materials were prepared at a powder-to-liquid ratio of 1:1, with complete mixing for 30 s at 23 °C (room temperature). Then, the materials were inserted into their respective matrices with a Centrix syringe (Maquira, Maringá, Paraná, Brazil) and pressed by a polyester strip. Specimens were stored at 37 °C for 24 h under 100% relative humidity. After this period, the specimens were removed from matrices.

### Microhardness test (*n* = 10)

Five indentations were performed on each specimen using 25 g load for 30 s (Ibrahim et al. 2017) with a minimum distance of 100 μm between each Vickers indentation (HMV-G series 21, Micro Vickers Hardness Tester, Shimadzu, Kyoto, Japan). Final hardness mean (in VHN) was calculated for each specimen.

### Sorption and solubility assay (*n* = 10)

Sorption and solubility tests followed 4049 ISO guidelines (2009). The specimens were stored in a desiccator with silica gel, and then incubated in an oven (Orion 520, Fanem, Sao Paulo, Brazil) at 37 °C for 22 h and in a desiccator at 23 °C for 2 h. Then, each specimen was weighed with a 0.01 mg precision analytical balance (ATX 224, Shimadzu, Japan) every 24 h until a constant mass was reached (m_1_).

Diameter and thickness were measured with a digital caliper (MPI/E-101, Mytutoyo, Tokyo, Japan) to calculate both area (mm^2^) and volume (mm^3^) of the specimen. The specimens were then individually and vertically immersed in 10 mL of distilled water for 7 days at 37 °C. After this period, specimens were rinsed with distilled water and dried with air spray for 15 s and weighed (m_2_) after 1 min.

Subsequently, the specimens were submitted to the same drying process described to obtain the (m_1_) until a new constant mass was obtained (m_3_).

Sorption and solubility (µg/mm^3^) were calculated by the following equations:

1 $${\text{Wsp}} = \frac{m2 - m3}{V},$$

2 $${\text{Wsl}} = \frac{m1 - m3}{V},$$

where Wsp corresponds to sorption; Wsl corresponds to solubility; *m*1 is the conditioned mass (before immersion in water); *m*2 is the mass (mg) after immersion in water for 7 days; *m*3 is the mass (mg) reconditioned; *V* is the volume of the specimen (mm^3^).

### Color change (Δ*E*) (*n* = 10)

According to ISO 7491 guidelines (2000), a color difference test was performed between the GIC-THEO and GIC-C groups. Using a spectrophotometer (CM3700A, Konica Minolta, Tokyo, Japan), specimen colors were recorded based on the CIELab colorimetric system. Color differences (ΔE) were determined by the following equation using a standard white background:

3 $$\Delta E = \left[ {\left( {L - L_{0} } \right)^{2} + \, \left( {a - a_{0} } \right)^{2} + \, \left( {b - b_{0} } \right)^{2} } \right]^{1/2} ,$$

where the values for *L*, *a*, and *b* parameters were obtained from GIC-THEO specimens and *L*_0_, *a*_0_, *b*_0_ parameters from the GIC-C.

### Antimicrobial ability (*n* = 20)

*Streptococcus mutans* biofilm quantification was based on previous studies (Waterhouse and Russell 2006; Nomura et al. 2009; Standar et al. 2010) with some amendments. GIC-C and GIC-THEO specimens were fixed with glue inside a 24-well culture plate. After sterilization with gamma radiation (25 kGy), *S. mutans* strains (UA 159), obtained from frozen stock collection, were activated on Tryptic Soy Agar (TSA, Difco, Leeuwarden, The Netherlands) at 37 °C for 16 h under microaerophilic/anaerobic conditions. After this period, the colonies were scraped and transferred to Tryptic Soy Broth (TSB, Difco, Leeuwarden, The Netherlands) medium until an optical density (OD_600_) of 0.3, detected by a spectrophotometer (Biophotometer, Eppendorf, Hamburg, Germany), was achieved. Then, colonies were stored under microaerophilic conditions for 3 h. All procedures were performed inside a laminar air-flow chamber under aseptic environment.

Then, the cultures were centrifuged at 5000×*g* for 5 min, and the supernatant was resuspended in Biofilm Medium (BM0) (Loo et al. 2000) plus 1% sucrose as a carbohydrate source to compose a standard microbial inoculum with OD_600_ = 0.8, approximately 1x10^10^ CFU/mL. Five aliquots of 1 mL were added to each well, and the samples were incubated under microaerophilic conditions at 37 °C for 3 h. Every 24 h, 1% sucrose enriched medium was replaced, over the course of 24, 48, 72 and 96 h time points.

For biomass evaluation, culture medium was removed, and the biofilm was statically stained with 500 μL of 0.4% safranin (Sigma, Gillinghan, England) for 15 min at room temperature in the presence of light. Specimens were rinsed three times by soaking in distilled water. Then, 500 μL of 95% ethanol was applied for 15 min to perform biofilm discoloration. After this period, five 50 μL aliquots of ethanol solution from each well were transferred to 96-well plates and assayed on an ELISA reader (Microplate Reader Model 680, Bio-Rad, Foster City, California, USA) at 490 nm (A_490_). Thus, five readings were obtained from each experimental well, in a total of one hundred readings.

### Fluoride release (*n* = 10)

Fluoride release test was performed as previously described (Carvalho and Cury 1999; Hayacibara et al. 2004; Nigam et al. 2009) with some amendments. The specimens were produced according to ISO 7489 standard guidelines (1986). Prior to the initial hardening of GIC-C and GIC-THEO, a piece of dental floss was added within the specimen so that it could be suspended in the solution.

After the initial sampling, specimens were removed from the matrices and placed in an oven (Orion, Fanen, Brazil) (37 °C, 100% relative humidity) for 24 h. Subsequently, each specimen was rinsed in 1 mL of deionized water and immersed in 1 mL of sterile artificial saliva [Ca 1.5 mM (CaCl_2_ 0.1665 g/L); 0.9 mM PO_4_ (NaH_2_PO_4_ 0.133 g/L); KCl 150 mM (KCl 11.184 g/L); Tris buffer 20 mM (2.4228 g/L) and 0.02% NaN_3_], and pH was buffered to 7.0.

Specimens were immersed in artificial saliva and stored in a shaker (Nova Ética, Model 430 RDB Incubator, São Paulo, Brazil) at 37 °C. Every 24 h, specimens were rinsed with 1 mL of deionized water and stored in 1 mL of artificial saliva, for 15 days. Control was performed on pure artificial saliva.

The fluoride electrode (Mettler-Toledo International Inc., Greifensee, Zurich, Switzerland, Model T50 Perfect ION Fluoride Combination) was previously calibrated with a series of five standard solutions (0.065, 0.125, 0.250, 0.500 and 1000 mg F^−^/mL) in triplicate. A 1:10 ratio of storage solution (TISAB III, total ionic strength adjustment buffer, Orion, Boston, MA, USA) was analyzed in duplicate. The results obtained per specimen were divided by the area of the specimen and expressed in μg/cm^2^.

### Statistical analysis

One-way ANOVA compared GIC-C and GIC-THEO regarding microhardness, sorption, solubility and color change (Minitab 17, Minitab Inc, Pennsylvania, USA), complemented by Tukey’s test for comparison, at 95% level of significance (*p* < 0.05).

Statistical evaluation of antimicrobial activity and fluoride release was performed by two-way ANOVA and Tukey’s test, at 5% significance level. For antimicrobial ability, the following variables were tested: GIC (GIC-C and GIC-THEO) and time points (24, 48, 72 and 96 h); for fluoride release, the factors were: GIC (GIC-C and GIC-THEO) and time points (1–15 days).

## Results

### Microhardness

ANOVA detected a statistically significant difference between GIC-THEO and GIC-C (*p* = 0.00), with increased microhardness for the GIC-THEO group.

### Sorption and solubility assay

ANOVA did not reveal a statistically significant difference between the values of sorption (*p *= 0.99) and solubility (*p *= 0.86) between the tested groups (Table 1).

**Table 1:** Microhardness, sorption, solubility results mean and standard deviation of experimental groups

Experimental groups	Microhardness (VHN)	Sorption (μg/mm^3^)	Solubility (μg/mm^3^)
GIC-THEO	60.96 ± 6.19 B	0.13 ± 0.036 A	0.018 ± 0.13 A
GIC-C	35.33 ± 3.90 A	0.13 ± 0.011 A	0.017 ± 0.003 A

Averages that do not share a letter are significantly different

### Color change

The color change (Δ*E*) between GIC-C and GIC-THEO was 1.38 ± 0.22, indicating that the addition of theobromine did not compromise the color of GIC.

### Fluoride release

**Fig. 1:**
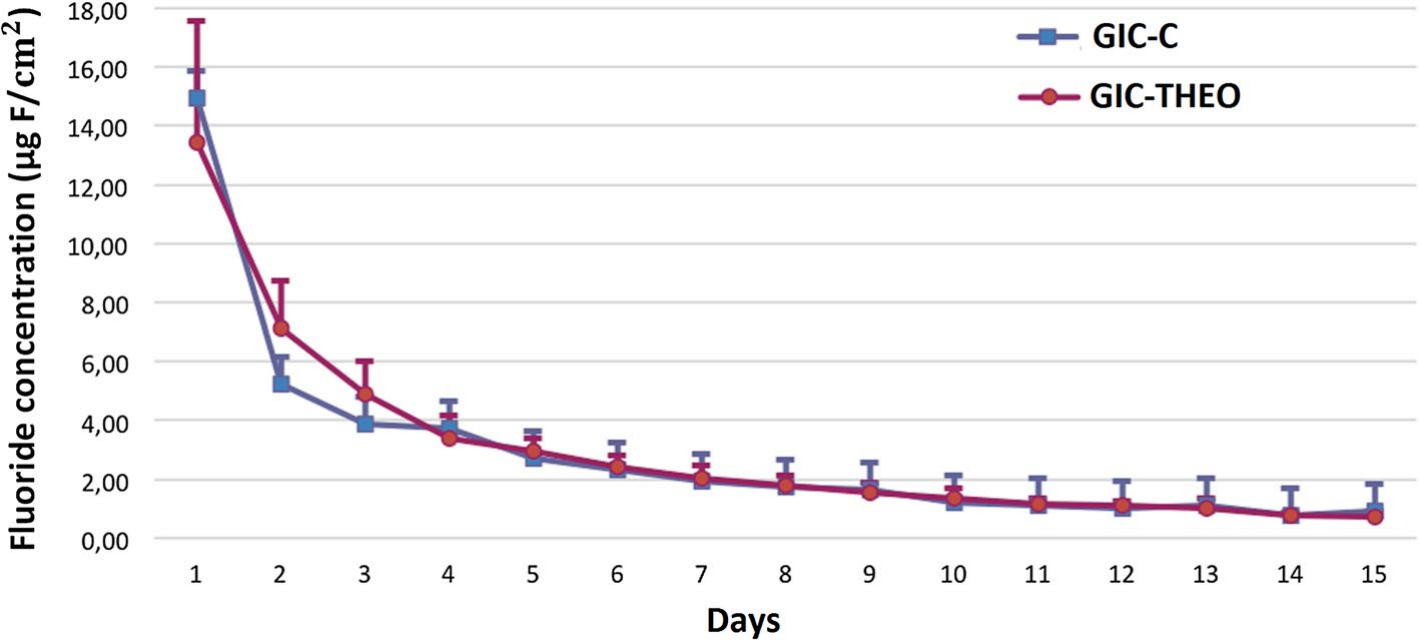
Fluoride dosage (μg F/cm^2^) in artificial saliva for GIC-C and GIC-THEO

Concerning fluoride release, ANOVA detected a statistically significant difference for tested time points (*p *= 0.00), while no statistically significant differences were detected for GIC (*p *= 0.529) and for variables interaction (*p *= 0.282). The addition of 1% theobromine did not compromise the fluoride release of GIC. Figure 1 clearly shows the reduction in fluoride released to saliva as a function of time, despite GIC tested. The concentration was strongly reduced from day 2 to 5, whereas the concentrations remained statistically similar from day 5 to 15.

### Antimicrobial ability

Regarding antimicrobial activity, ANOVA detected a statistically significant difference for time points (*p *= 0.001) and GIC (*p *= 0.005). However, the interaction between these factors was not statistically significant (*p *= 0.152). Regarding time points, the lowest amount of biofilm was deposited after 24 h, while the largest amount of biofilm was deposited after 96 h. Intermediate amounts of biofilm were detected at 48 and 72 h, with no statistically significant difference between them. Lower amounts of biofilm were detected in GIC-THEO samples than in GIC-C, indicating that the inclusion of 1% theobromine increased the antimicrobial ability of GIC.

## Discussion

In this study, the null hypothesis was partially accepted, since some properties of GIC were modified by the addition of theobromine. This is the first study that added theobromine to a dental cement, but this alkaloid has already showed positive results as a remineralizing substance added to mouth rinses and dentifrices (Matsumoto et al. 2004; Kargul et al. 2012; Gazzani et al. 2012; Badiyani et al. 2013; Amaechi et al. 2013, 2015; Lippert 2017), providing the rationale for this study.

The literature reports opposing results with respect to the active component added to GIC. There was a considerable decrease in Vickers hardness when GIC was previously immersed in buffer medium containing acidulated fluoride phosphate (AFP) and TiF_4_ (Topaloglu-Ak et al. 2012) or when adhesive/nanofiller monomers were added to GIC (Bagheri et al. 2013). On the other hand, due to the incorporation of *N*-vinylcaprolactam (Moshaverinia et al. 2011) into conventional glass ionomers, no hardness modification was detected.

GIC-THEO results showed an increased microhardness, when compared to GIC-C, in accordance with another study that added proline and 0.1% epigallocatechin-3-gallate (EGCG) to a conventional GIC (Ansari et al. 2013; Amaechi et al. 2013). Researchers (Hu et al. 2013) suggest that by adding EGCG to GIC, an increased chelation between phenolic hydroxyl and carboxyl groups in GIC occurred, leading to an enhanced formation of polysaccharides and crosslinks, modifying the material into a more complex structure. In addition, gaps between GIC particles can be reduced if a higher acid amount reacts with the powder of GIC. These modifications together increased the amount of molecular bonds on the surface of the material, leading to an increase in microhardness (Hu et al. 2013; Zoergiebel and Ilie 2013). As theobromine also belongs to the family of polyphenols, we suggest that the addition of theobromine to the GIC may exert a similar effect as the inclusion of EGCG with regard to microhardness outcomes.

Sorption and solubility assays detected similar results for GIC-THEO and GIC-C. However, other studies detected higher water sorption and greater microleakage when other substances such as 1% natural propolis, 2% lyophilized propolis (Troca et al. 2011), or 0.06, 0.08 and 0.1% per weight of silica particles (Felemban and Ebrahim 2016) were utilized in modified GICs. In these studies, (Troca et al. 2011; Felemban and Ebrahim 2016), compressive and tensile strengths were preserved. It is fundamental to consider that propolis, a natural resin-like material, and silica particles are primarily focused on the context of increasing material strength, but this focus should not compromise other properties. Additionally, the addition of 2.5% of chlorhexidine diacetate with 2.5% of cetrimide yielded an increased solubility of GIC (Korkmaz et al. 2013). Therefore, since the result of the chemical reaction of the GIC with theobromine did not change the sorption and solubility of the material, if sufficient additional properties are not compromised, its use could be considered safe.

The addition of theobromine did not compromise the color of GIC, as a study that evaluated the addition of chlorhexidine (CHX) and resin to GICs (Prabhakar et al. 2013) observed a greater color stability for materials modified by CHX than by resin. Literature indicates that values of ΔE lower than 1 are considered non-perceptible to the human eye, while values between 1.1 and 3.3 are clinically acceptable (Zhou et al. 2018). Thus, our results are considered favorable in support of the innovation proposed in this manuscript, as the slight color change presented by GIC-THEO is clinically acceptable and does not compromise its application.

In relation to the amount of biofilm formed by *S. mutans*, a considerable decrease in biofilm was detected in GIC-THEO samples. Similar results were observed when the inclusion of 0.1% epigallocatechin-3-gallate (EGCG) was tested in a conventional GIC, with a reduction in the amount of biofilm formed on the modified GIC specimens (Hu et al. 2013). It is suggested that the antibacterial action of theobromine may be related to additional factors, such as the reduction of bacterial adhesion (Percival et al. 2006; Hu et al. 2013; Sudharsana and Srisakthi 2015), inhibition of glucosyltransferase enzyme secreted by cariogenic bacteria, which reduces biofilm formation and acid production of *S. mutans* (Hu et al. 2013), suppression of saliva and bacterial amylase activity, causing alteration in carbohydrate metabolism, which reduces the rate of acid production by *S. mutans* or causing irreversible damage to the cytoplasmic membrane of bacteria (Matsumoto et al. 2004). Thus, we consider that the addition of theobromine empowered the antimicrobial effect of GIC.

Fluoride release after the addition of 1% theobromine to GIC did not change, as similarly observed when 0.1% epigallocatechin-3-gallate (EGCG) (Hu et al. 2013) was added to GIC. As both of these active components are from the polyphenol family, we suggest that a strong anti-cariogenic action could be a positive attribute of the use of GICs.

Since the solubility was not changed for GIC-THEO, the fluoride ion release for the medium curve meets that of GIC-C, with higher release rates in the first 24 h (Prabhakar et al. 2013) and a reduction over the storage period, and with a tendency to stabilize after 5 days. An additional study (Amaechi et al. 2013) reported that this stabilized release occurs due to a decrease in the concentration of the carboxyl groups (COOH).

The limitation of this in vitro study mainly consists of the absence of similar studies to challenge and corroborate the obtained results. In this sense, as theobromine and EGCG are each substances from the polyphenol family, we speculated that correlating our results with EGCG could be an interesting approach. Thus, it can be considered that the incorporation of 1% of theobromine into a conventional GIC can represent an interesting strategy to enhance microhardness and antimicrobial effects without compromising other tested properties. The stability of the improvements observed herein should be verified in longitudinal studies. It is important to note that we used a commercial GIC (GCGold Fuji 9, GC Corp, Japan) and a commercial theobromine compound (Sigma-Aldrich, Darmstadt, Germany) to compose our modified product and that no further characterization of the material was performed. Thus, our results are valid for the combination of these two products.

## Conclusion

Because the addition of theobromine to GIC increased microhardness and reduced the amount of biofilm formed over the material surface, did not interfere with sorption, solubility, color; and fluoride released to saliva, it can be considered a valid alternative approach to improve the performance of GIC.
